# Adding rewards to regulation: The impacts of watershed conservation on land cover and household wellbeing in Moyobamba, Peru

**DOI:** 10.1371/journal.pone.0225367

**Published:** 2019-11-20

**Authors:** Javier Montoya-Zumaeta, Eduardo Rojas, Sven Wunder

**Affiliations:** 1 Crawford School of Public Policy, Australian National University, Canberra, Australia; 2 Center for International Forestry Research (CIFOR), Bogor, Indonesia; 3 Faculty of Geography and History, University of Barcelona, Barcelona, Spain; 4 European Forest Institute (EFI), Barcelona, Spain; Ghent University, BELGIUM

## Abstract

We estimate the effects of Peru’s oldest watershed payments for environmental services (PES) initiative in Moyobamba (Andes–Amazon transition zone) and disentangle the complex intervention into its two main forest conservation treatments. First, a state-managed protected area (PA) was established, allowing sustainable use but drastically limiting de facto land use and land rights of households in the upper watershed through command-and-control interventions. Second, a subset of those environmentally regulated households also received incentives: PES-like voluntary contracts with conditional in-kind rewards, combined with access to participation in sustainable income-generating activities of the integrated conservation and development project (ICDP) type. To evaluate impacts, we perform matching procedures and adjustment regressions to obtain the average treatment effect on the treated (ATT) of each intervention. We investigate impacts on plot-level forest cover and household welfare for the period 2010–2016. We find that both treatments—command-and-control restrictions and the incentive package—modestly but significantly mitigated primary forest loss. Incentive-induced conservation gains came at elevated per-hectare implementation costs. We also find positive effects on incentive-treated households’ incomes and assets; however, their self-perceived wellbeing counterintuitively declined. We hypothesise that locally frustrated beneficiary expectations vis-a-vis the ambitiously designed PES-cum-ICDP intervention help explain this surprising finding. We finalise with some recommendations for watershed incentives and policy mix design in Moyobamba and beyond.

## Introduction

Learning processes regarding conservation incentives have often been based on anecdotal evidence [1, 2], rather than on rigorous impact assessments [3, 4]. Randomised control trials have been almost absent in testing conservation incentives, barring some recent exceptions [5]. Other rigorous empirical studies have also been scarce and, in the Southern Hemisphere, heavily concentrated on Costa Rica and Mexico [6–8]. However, the number of published quasi-experimental studies has recently expanded [9].

Peru is an illustrative case in point. Historically, the typical policy response to the deforestation and degradation of the Amazon rainforest has been to establish protected areas (PAs). About one-quarter of the Peruvian Amazon is under a state-managed protection regime [10]. Conservation incentives have been piloted only in a few cases, experimenting with design modalities [11]. Recent evidence suggests marginally positive conservation effects [12–14], including those from complementary policies such as communal land titling [15]. Policy impacts on local populations’ wellbeing remain even less clear [13].

Here we aim to contribute to filling the empirical gap regarding Peruvian conservation incentive programs by analysing one prominent conservation initiative, the Moyobamba Reward for Hydrological Ecosystem Services Mechanism—Peru’s pioneer watershed-based payment for environmental services (PES) initiative [16, 17]. Our objective is to quantify the effects of this initiative’s different treatment components on plot-level land cover changes, as well as the effect of conservation incentives on treated participant households’ wellbeing. We proceed as follows: in the next section, we review the literature using (experimental and quasi-experimental) robust methods to evaluate PES impacts in developing countries. Next, the Peruvian watershed intervention is discussed. After that, we describe in detail the study area and our analytical methods. We then present our results and finally consider them in a wider perspective.

### Evidence on impacts of PES-like interventions

Direct payments conditioned on environmental performance of voluntarily contracted agents have recently been considered an appealing way to address threats to ecosystems and their services [18, 19]. Further, synergies between these type of interventions, widely known as PES, and wellbeing-related benefits have also been claimed (e.g., [20, 21]), thus making PES a particularly interesting possibility for adoption in developing countries.

Much of the PES debate so far has discussed concepts and provided qualitative descriptive analyses of limited cases [6]. Rigorous impact assessments are scarce, as in the field of conservation in general [7, 9, 22]. This is even more the case for socio-economic impacts [6]. We summarise findings from studies evaluating the environmental and socio-economic impacts of PES in S1 Table. Geographically, much analysis has been concentrated in Costa Rica [23–25], with the national PES program there being the first of its kind [26]. Follow-up nationwide PES programs in Mexico [27–32], China [33, 34], Ecuador [35, 36] and Peru [12] have also been impact evaluated.

Notably, some studies detected very low forest impacts in the early implementation phases of national programs [12, 23, 27], yet found higher impacts from the same programs in subsequent stages [24, 28, 29]. Arguably, the latter also used analytical techniques that better captured heterogeneous impacts and identified causal mechanisms more accurately [37].

Livelihood-oriented PES evaluations have come mostly from Asia. For instance, PES notably accelerated livelihood transitions among rural Chinese households [33, 38]. Clements and Milner-Gulland [39] demonstrated that PES in two Cambodian PAs significantly reduced poverty incidence, raised agriculture revenues and increased food security, allegedly due to synergies between conservation instruments (PES and PA); effectively excluding outsiders allowed resident households to sustainably use available forest and land resources to their own benefit. In Latin America, no significant wellbeing impacts on national-level PES participants were found from 1996 to 2005 in Costa Rica [25], nor from 2007 to 2013 in Mexico [32]. Nevertheless, a recent analysis found positive impacts of a local hydrological PES scheme on participant households’ assets enrolled through communal agreements in Chiapas, Mexico [40].

### The case of Moyobamba

Construction of the interregional Carretera Marginal highway effectively promoted economic integration between the coastal and Amazonian regions of Peru but also triggered land clearing, forest loss and degradation through spontaneous settlements alongside the highway, mainly by colonists from neighbouring departments [41]. In the San Martin Department, one of the deforestation hotspots has been around Moyobamba. Therefore, in 2004, local authorities established a municipal conservation area, mainly to protect the city’s drinking water sources through traditional command-and-control measures [16].

The Moyobamba watershed protection initiative began in 2004, as part of the Cuencas Andinas Regional Project, which also features innovative, incentive-based models of integrated watershed management in several pilot sites across the Andean region [42]. The initiative achieved the participation of key stakeholders such as the *Empresa Proveedora de Servicios de Saneamiento* (EPS) Moyobamba (the public supplier of local drinking water) and the *Proyecto Especial Alto Mayo* (PEAM), a long-term state-funded program promoting productive entrepreneurship in the Alto Mayo watershed. From 2004 to 2006, hydrological assessments and PES feasibility studies were undertaken, including collating socio-economic diagnostics of the population settled in the upper watersheds and identifying Moyobamba drinking water users’ willingness to pay for improved watershed regulation services [43].

**Fig 1:**
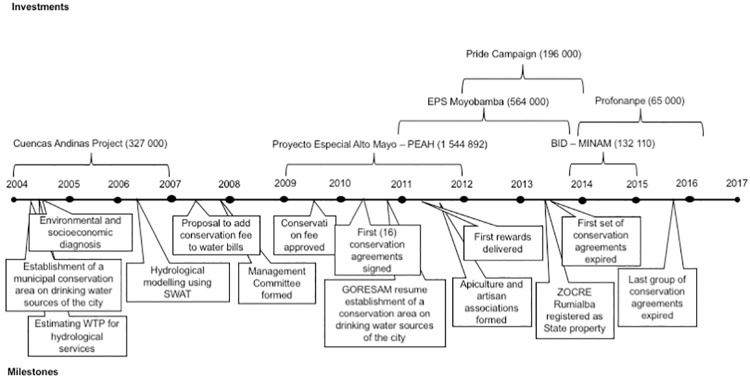
Timeline of project interventions, 2004–2017. Above the timeline are labelled the project funding activities of the initiative; invested amounts (in parentheses) are in Peruvian *soles* (PEN). Source: own elaboration, based on MINAM [47].

Subsequently, a PES-type intervention was designed, primarily to address increasing costs for EPS Moyobamba associated with an observed 20% acceleration of sediment loads between 2003 and 2005, which was being related to upstream land use changes [44]. Hydrological modelling identified the necessity to prioritise efforts in the Mishquiyacu and Rumiyacu micro-watersheds, maintaining remaining forest cover around water catchments and establishing agroforestry systems on already established coffee plots and treeless pasturelands to decrease soil erosion and stabilise dry season water flows [44, 45]. The third upper watershed, Almendra, was much less degraded, yet was included preventively [41]. By 2009, EPS Moyobamba had managed to raise modest PES funding from local water bills, adding a monthly PEN1.00 (approximately USD0.32) user fee. Simultaneously, a management committee was formed, representing EPS and other local organisations, to account for accumulated contributions collected through the water bills and to formulate and implement adequate investment projects. Fig 1 provides a timeline for the activities of the project since 2004, including also indicative amounts of resources invested into the initiative over time. Importantly, collected funds from water users have never sufficed to cover the envisaged investments: only PEN85,632 (USD25,793) were collected yearly [46]. This represents only about one-fifth of the average annual investments from 2009 to 2014. Hence, supplementary funding from public and private sources has continuously been required.

Aligned with the state norms for use of public funds, the management committee established that rewards to participant farmers should be in kind, rather than cash, allegedly so as not to compromise accountability [16]. Rather than handing over consumption goods, the idea was to provide distinct investments for farmers, which would allow them to improve their livelihoods, diversify income sources and simultaneously take actions for forest conservation, reforestation and sustainable use. This jointly would limit erosion (and thus sedimentation in water courses) while also stabilising dry season flows. The combined set of activities can best be described as an integrated conservation and development program (ICDP) [48, 49]. In practice, significant emphasis was placed on shifting traditional treeless coffee farming into shade-based agroforestry coffee systems, diversifying into new on-farm sustainable income sources and providing assets to raise living standards. In principle, the intervention was designed as a conditional ICDP, combined with conditional land rights; both support elements could, in principle, be discontinued in case of environmentally noncompliant actions.

Consequently, three type of investments emerged: 1) enhancing on-farm tree cover through agroforestry, reforestation and tree fences, 2) improving physical infrastructure (e.g., rain collector systems, ecological toilets, coffee post-harvest processing equipment) and 3) promoting new income-generating activities (e.g., apiculture, guinea pigs, handicrafts). These in-kind rewards, conditional upon conservation contract signature and continuous compliance, were customised to each household’s requirements [16]. From 2010 to 2012, 59 plots located in the priority area were enrolled in the initiative, after signing conservation agreements with their 49 land managers for conditional in-kind rewards funded by PEAM, stretching mostly over three years [50]. However, after this period, rewards became more irregular and subject to less available project funding, thus affecting successive renewals and contracting of new PES agreements. Seemingly, a new five-year public investment project formulated by EPS will be implemented soon.

Parallel to the design and execution of the PES-like scheme, since 2010, the Regional Government of San Martin (GORESAM) reinforced attempts to enforce land use restrictions in drinking water sources for Moyobamba, establishing the Rumialba Ecological Recovery and Conservation Zone (ZOCRE). This command-and-control measure was implemented to counteract risks of latent disordered occupation of the zone, including land trafficking [51]. The land was publicly registered as state property in 2013, but responsibility for its management was delegated to the Regional Environmental Authority, an office reporting directly to the GORESAM [52]. However, the ZOCRE could not yet be included in the nationwide PA system, which somewhat curbed enforcement potentials.

**Fig 2:**
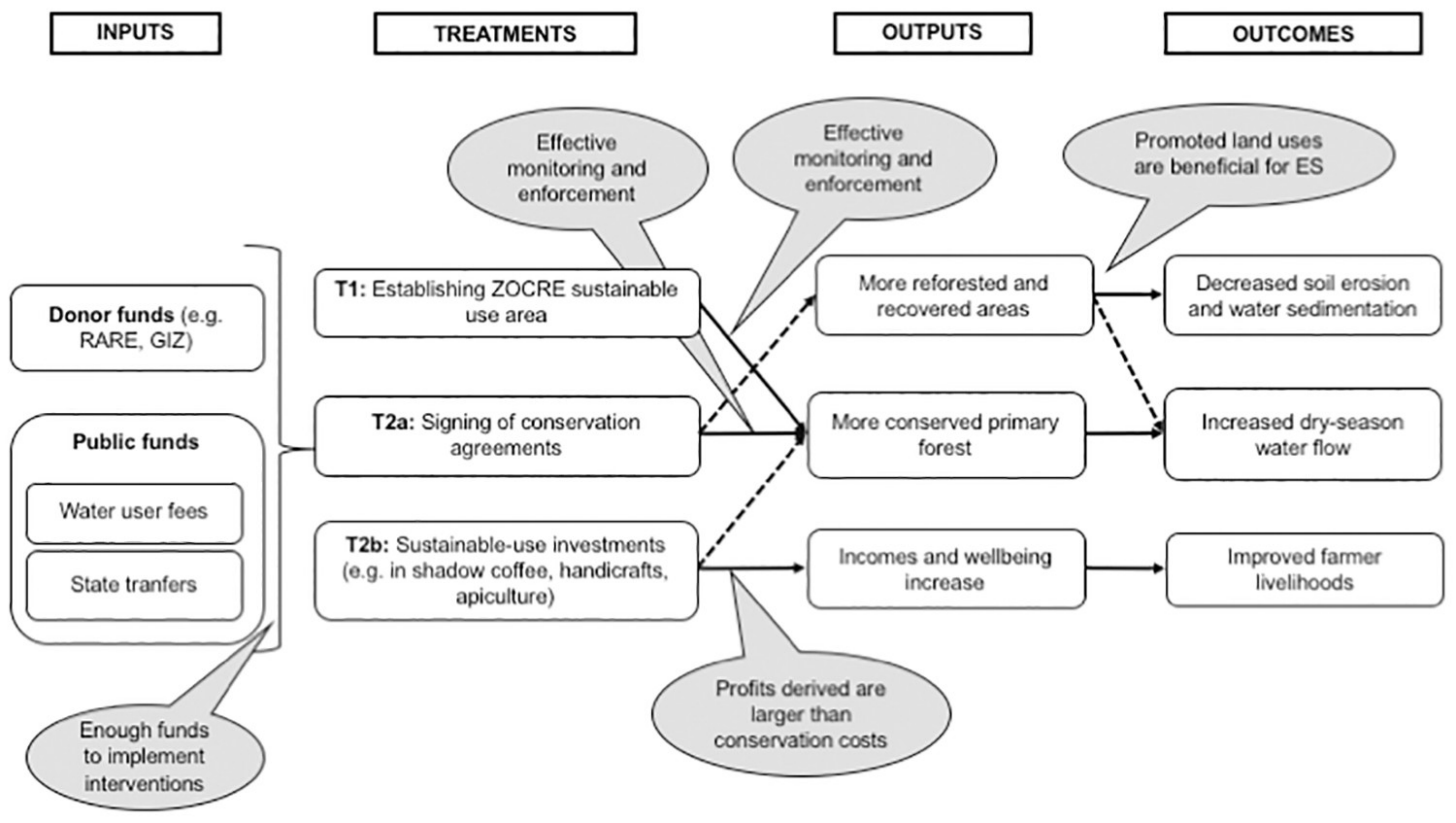
Theory of change for the Moyobamba watershed initiative. Source: own elaboration.

We ask the following question: how did the initiative’s implementers intend to achieve the desired conservation impacts on the ground? Summarising their variable actions into a theory of change (see Fig 2), we characterise two allegedly synergetic treatments for our analysis:

- **Treatment 1 (T1): command and control.** This treatment involved the declaration of a state-managed conservation use area (ZOCRE Rumialba), limiting de facto land rights in plots fully or partially overlapping the ZOCRE Rumialba and its influence zone (as mapped below).
- **Treatment 2 (T2): PES–ICDP mix.** This treatment involved the provision of incentives to households managing plots located in the prioritised upper watersheds, combining a) in-kind, allegedly conditional rewards delivered directly to voluntarily contracted households, b) granting of temporal land rights kept conditional on benign land uses and c) access to participation in sustainable economic activities.

From 2010 (our baseline year), approximately 140 plots were treated with T1, while 59 plots in total were treated with T2 [50]. The T2 treatment implied potential benefits to households but the stakes were also raised: monitoring would be more frequent, and in case of participants being caught in environmental noncompliance of contracts, sanctions would be harsher than for households in the ZOCRE that had received no incentives. We can, therefore, state that receiving incentives was accompanied by a greater threat of more severe command-and-control-induced sanctions, equalling a greater treatment intensity of the entire policy mix (T1 + T2).

## Material and methods

### The study area

**Fig 3:**
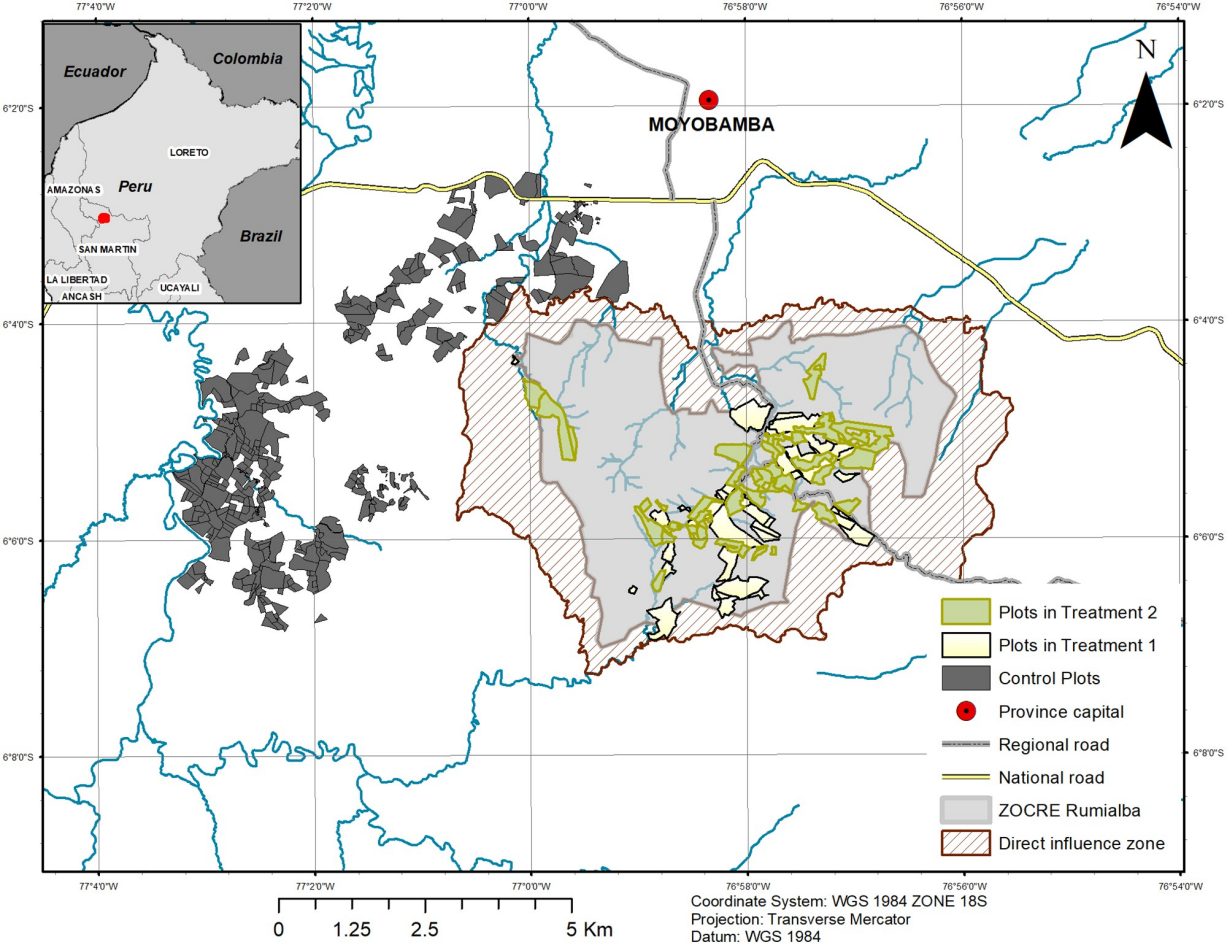
Sampling of treated and control plots: ZOCRE Rumialba (T1) and PES area (T2). Source: own elaboration, based on publicly available data from EPS Moyobamba, PEAM, GORESAM and the Ministry of Transports and Communications.

The province of Moyobamba is located in the Department of San Martin, in north-western Peru, between the highland and Amazon regions (see Fig 3). The ZOCRE Rumialba covers 2396 ha, including the upper micro-watersheds of the Rumiyacu, Mishquiyacu and Almendra rivers. These provide drinking water to Moyobamba (about 50,000 inhabitants) [53]. Predominant natural land cover is premontane tropical forests, according to the Holdridge life zones system; temperatures fluctuate between 20° C and 24° C. Average annual precipitation is 1600 mm, with a marked rainy season between November and May and a drier season from June to August [16].

Moyobamba province received significant migratory influxes since the 1970s, with the construction of the Carretera Marginal; these have levelled off since 2007. From 1981 to 2002, however, annual population growth averaged 5.5%, mainly due to immigration from nearby departments such as Cajamarca, Amazonas, Piura and Lambayeque. Traditionally, the region produces dryland rice (in flat parts of the Alto Mayo river basin), cattle and coffee (on hillsides). Annual deforestation in the Alto Mayo region thus rose to 4.2% in the early 2000s [44]. Moyobamba has the most productive coffee lands in the San Martin Department [54–56]. The ZOCRE Rumialba is located at 6 km from central Moyobamba and is currently inhabited by around 200 migrant households of mostly Andean origin. Their cash income sources include crops (mainly coffee), livestock and off-farm employment [57].

### Sampling and data collection

One of the first challenges we faced regarded identifying adequate control households or plots to compare with treatment groups; all comparable local upland areas were already or were in process of becoming state protected. We could, therefore, either opt for biophysically similar control plots (e.g., in terms of size, slope or altitude) from other regions but with different local development dynamics or for biophysically different plots from the same Moyobamba region. We adopted the second approach and then used statistical methods (further described below) to make the impact assessment possible.

Considering the above-mentioned challenge, our sampling procedure was performed in three steps. First, we merged cartographic shapes provided by PEAM, EPS Moyobamba and GORESAM to delimit the study area for the baseline year (2010). This allowed us to obtain a complete list of plots under treatments T1 and T2, respectively, and to identify non-treated control plots located in surrounding sectors of the ZOCRE.

Second, we conducted a total of 311 households surveys. Although we intended to include all 49 conservation agreement signatories (T2), we had to disregard seven of them: four were not households but instead were institutional representatives (e.g., a school or an non-governmental organisation) and thus not comparable, two households declined to participate in the study and one had a plot not located inside the prioritised area. We also surveyed 40 randomly selected households in the ZOCRE and its influence zone (T1), which accounts for 29% of all those available, given budget and time restrictions. For the non-treated control group, we selected 10 sectors with similar geographical and biophysical features, obtained lists of plots linked to these and then randomly selected and surveyed 229 households. This number exceeds the minimal sample size (estimated at 196 with 5% maximum allowable error for an identified population of 399 non-treated households in areas surrounding the ZOCRE). We restrict our analysis below to plots for which we could obtain both household surveys and remotely sensed land cover data (see Fig 3).

For our land cover analyses, we used geographic information system tools to estimate variables such as average slope, altitude, distance to Moyobamba and land cover changes at plot level, for which we used 2005, 2010 and 2016 Landsat imagery with a resolution of 30 m per pixel, respectively. The images allowed us to estimate, with relatively high precision, land cover changes in two periods: before treatment (from 2005 to 2010) and during the treatment period (2010–2016). RapidEye imagery with higher resolution (5 m per pixel), which was only available for the year 2011, was used complementarily to guide image interpretation. To classify plot-level land cover and its changes, we followed Potapov et al.’s [58] definition of forests as ‘areas with trees above 5m and tree canopy cover above 30% within Landsat 30m pixels’. We differentiated between primary and secondary forests, based on the visible signs of recent human alteration that are present in the latter.

This study met with the ethical standards established in the Latin American and Caribbean Environmental Economics Program’s (LACEEP) grant contract IDEA-186/2016. Our ethics protocols were approved by the Human Research Ethics Committee of the Australian National University (Human Ethics Protocol 438/2018), which included the explicitly documented free prior informed consent of all participants and measures to maintain the confidentiality of the data.

Household surveys were conducted in March and April 2017 by four previously trained enumerators and addressed questions about demographics, agricultural practices, assets and income. They also included a procedure to record Universal Transverse Mercator (UTM) coordinates in plots to validate our cartographic information. As in similar studies (e.g., [25, 32, 40]), we used recall questions to establish pre-intervention covariates, given the unavailability of information at this disaggregated level. The first author consulted regarding the content of the draft questionnaire with key institutional stakeholders (e.g., EPS Moyobamba, the management committee, PEAM, German Cooperation-GIZ, GORESAM) and then piloted the revised questionnaire in two rounds (15 and 30 surveys, respectively) in two nearby districts (Jepelacio and San José de Sisa) before finalising the survey instrument.

**Fig 4:**
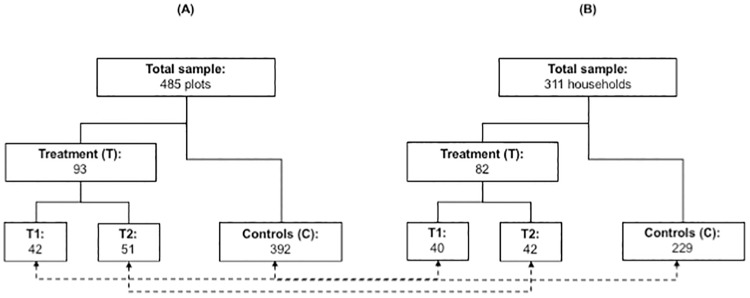
Composition of plot- and household-level datasets. Dotted arrows represent that households included in the dataset (B) manage plots in the dataset (A).

Subsequently, we built two datasets (see Fig 4). First, the plot-level land cover dataset (A) contains geographical and biophysical data of treated and non-treated plots. Second, the household-level dataset (B) contains biophysical, demographic and socio-economic information for all the households managing sampled plots in (A).

### Empirical strategy

For 2010 to 2016, we evaluate the effects of both treatment components (command and control and the PES–ICDP mix) of the Moyobamba initiative on primary and secondary forest cover at the plot level. Likewise, we also estimate the simultaneous effects on the following wellbeing outputs:

- **Household yearly incomes**—aggregated self-reported agricultural, environmental and off-farm incomes for the years 2010 and 2016 from the survey.
- **Household asset holding**—a 0–11 scaled index based on the sum of self-reported assets, indicating a status of material wellbeing (e.g., vehicles, motorcycles, cell phones, cattle, gas stove) for the years 2010 and 2016.
- **Quality of life:** households’ self-reported answers to the survey question: do you consider that the quality of life of your family compared with 2010 is the same, has improved or worsened?

For each indicator, we again estimated the average treatment effect on the treated (ATT) observations:

$$\text{ATT}=\text{E}\left({\text{y}}^{1}–{\text{y}}^{0}|\text{D}=1\right)$$

where y^1^ denotes the output under the treatment, y^0^ denotes the same output in the absence of treatment and D is a dummy variable that takes on the value of one when the unit of observation has been treated and takes on the value of zero otherwise. The key assumption is that control and treatment groups are sufficiently similar (at least in terms of observable covariates) and are, therefore, comparable [37]. Hence, we first identified potential confounders that need to be controlled for. Based on similar studies (e.g., [24, 25, 35, 39, 59]) and information from key stakeholder interviews, we selected a set of eight covariates for our land cover analysis and nine for the wellbeing assessment. Then we measured how much treated observations differed from their corresponding controls.

This first so-called naïve comparison between unmatched treatments and control plot reveals significant differences in terms of practically all covariates; the same conclusion arises from the comparison between households in the treatment (T1, T2) and control groups. This is unsurprising, since T1, at least, had been focused on the entire drinking water watershed sources of Moyobamba city, an area featuring geographically and biophysically different characteristics, such as greater altitude and slope, compared with the surrounding landscape (see Table 1). Further, plots under both treatments are larger, had larger forest coverage in the baseline year (2010) and also demonstrated a higher pre-treatment increase in secondary forests (2005–2010).

**Table 1:** Summary statistics for land cover on plots.

*Variable*	(1)	(2)	(3)	(4)	Differences^a^	
	Total *n = 485*	Treat. 1 *n = 42*	Treat. 2 *n = 51*	Not treated *n = 392*		
					T1–C	T2-C
	Mean (SD)	Mean	Mean	Mean	(2)–(4)	(3)–(4)
***Outcomes (2010–2016)***						
Δ Primary forest (ha/plot)	–0.039 (0.19)	–0.048	–0.02	–0.041	–0.007	0.02
Δ Secondary forest (ha/plot)	–0.019 (0.36)	0.019	–0.045	0.027	–0.008	–0.072*
***Covariates***						
Plot size (ha)	2.90 (4.51)	5.45	4.28	2.45	3.00***	1.83***
Slope (degrees)	6.31 (6.01)	16.1	14.32	4.21	11.88***	11.11***
Altitude (m)	890.7 (80.99)	980.98	992.1	867.84	113.1***	124.25***
Distance to Moyobamba (km)	8.41 (3.08)	9.64	8.27	8.30	1.34***	–0.03
Forest cover 2010 (ha)	0.88 (1.99)	2.28	1.90	0.60	1.67***	1.29***
Δ Primary forest 2005–2010 (ha/plot)	–0.052 (0.21)	–0.168	–0.068	–0.037	–0.13***	–0.031
Secondary forest 2010 (ha)	1.15 (1.86)	2.42	1.86	0.93	1.49***	0.93***
Δ Secondary forest 2005–2010 (ha/plot)	0.195 (0.92)	0.567	0.577	0.106	0.46***	0.471***

Note:

^(a)^ Statistical differences are evaluated using a t-test and reported at 10%*, 5%** and 1%*** significance levels.

In addition to these biophysical differences, treated households initially had less income and fewer assets (see Table 2), in line with claims that incentives were mainly being targeted at poorer households.

**Table 2:** Summary statistics for the household welfare dataset.

*Variable*	(1)	(2)	(3)	(4)	Differences^b^	
	Total (*n = 311*)	Treat. 1 (*n = 40*)	Treat. 2 (*n = 42*)	Not treated (*n = 229*)	T1–C	T2–C
	Mean (SD)	Mean	Mean	Mean	(2)–(4)	(3)–(4)
***Outcomes***						
Δ Yearly household income (in PEN)	419 (2392)	970	1279	164	805**	1115***
Δ Assets index	1.18 (1.44)	1.33	1.88	1.03	0.3*	0.85***
Improved life quality (0/1)	0.67 (0.47)	0.63	0.36	0.73	–0.10*	–0.37***
Worsened life quality (0/1)	0.14 (0.34)	0.10	0.29	0.11	–0.01	0.18***
***Covariates***^a^						
Total size of managed lands (ha)	4.79 (6.33)	6.69	6.24	4.19	2.49**	2.05**
Average slope (degrees)	7.31 (6.38)	16.2	14.48	4.44	11.76***	10.04***
Average altitude (m)	885.79 (66.86)	912.08	936.21	871.95	40.12***	64.26***
Average distance to Moyobamba (km)	8.20 (3.19)	9.74	8.25	7.92	1.81***	0.32
Total forest cover 2010 (Ha)	3.43 (4.63)	5.80	5.59	2.62	3.18***	2.97***
Δ Total forest 2005–2010 (ha/hh)	0.24 (1.14)	0.49	0.65	0.12	0.37	0.53***
Household members 2010	2.94 (1.13)	3.08	2.74	2.95	0.13	–0.21
Assets index 2010	3.31 (1.98)	2.63	2.40	3.59	–0.96***	–1.19***
Income 2010 (PEN)	15,383 (16,681)	8514	9490	17,664	–9150***	–8174***

Note:

^(a)^ Here we nested plots at household level: plot size, total forest (2010) and total forest change (2005–2010) were added, while slope, altitude and distance to Moyobamba were averaged among the number and size of plots per household.

^(b)^ Statistical differences are evaluated using t-test and reported at 10%*, 5%** and 1%*** significance levels.

To reduce these significant differences between groups, we first implemented statistical matching techniques, as used in other conservation impact evaluations [37]. We identified and matched observations in the control group that were similar to treated observations in terms of their baseline covariates. Matched observations comprised the subgroup that served as reference to estimate the effect of the treatment on outcomes and constituted the counterfactual (i.e., the scenario that hypothetically would have happened had the intervention not been implemented) [25].

We performed three matching algorithms: 1) 1-to-2 nearest neighbours (NN) with replacement using the Mahalanobis distance (M2NN), 2) kernel propensity score (K-PS) matching using 0.05 caliper and 3) radius propensity score (R-PS) matching, also with 0.05 caliper. To measure the effectiveness of each algorithm to reduce bias, we used as criterium the standardised percentage difference between means ($\%St.Dif.=\frac{{\overset{-}{X}}_{T}-{\overset{-}{X}}_{c}}{\sqrt{\frac{\left(S_{T}^{2}+S_{C}^{2}\right)}{2}}}$, where *T* and *C* represent treated and control groups, respectively) for each covariate, considering the usual rule of > 25% to determine presence of persistent bias [60]. All three matching algorithms significantly reduced bias in covariates, but the two propensity score–matching algorithms achieved much more balanced panels (see S2 Table), at the expense of dropping off some treated observations that fell outside the propensity score’s common support level. In the case of K-PS, the matching procedure discarded 15 (36%) plots and 21 (53%) households from the T1 group, and 13 (25%) plots and 17 (40%) households from the T2 group. Likewise, R-PS matching discarded 16 (38%) plots and 24 (60%) households from the T1 group, and 13 (25%) plots and 17 (40%) households from the T2 group. We used the user-written package psmatch2, available for Stata 14.0 [61], to perform all described matching procedures. After each matching, we ran multivariate regressions with resulting matched samples to minimise remaining bias and estimate the adjusted treatment effect [62].

We also reported Rosenbaum bounds (Γ) after each post-matching mean comparison between treatment and control groups, to assess unobserved heterogeneity that could potentially undermine the statistical significance of the evaluated treatment, as in other impact evaluation studies [13, 24, 36]. We repeated this for all environmental and wellbeing effects that presented statistic differences of 10% or greater [63]. Additionally, we estimated the intracluster correlation for all the outcomes, considering sectors as the cluster unit to measure potential spillover effects [64]. These results are shown in S3 Table.

Finally, our reading of the literature does not identify any of the three matching methods as superior in absolute terms; each has pros and cons. As such, we report their results simultaneously, also displaying transparently some sensitivity of the impact evaluation results vis-a-vis the adopted method and the size of the included sample. While these factors do not call into question the overall direction of our conclusions, the size and statistical significance of a few results is clearly sensitive; therefore, it is preferable to present them using ranges, rather than point estimates.

## Results

### Effects on plot-level land cover

We started our analysis comparing plots under T1 (command-and-control measures) versus controls plots. The comparison in Table 2 shows that plots under this treatment were on larger than average controls, thus holding more primary and secondary forests. The matching techniques allowed us to significantly reduce these biases for the land cover analysis (see S2 Table). We summarise the estimated land use effects of both treatments in Table 3. For T1, both K-PS and R-PS matching algorithms yielded significant results, while the M2NN did not, failing to produce balanced covariates vis-a-vis the aforementioned biases. We thereby confirmed significant (5% level) positive effects of T1 on mitigating primary forest loss, ranging from 0.11 (after R-PS matching) to 0.14 ha/plot (after K-PS matching); for M2NN, the coefficient is positive yet insignificant at 10% (p = 0.106). Treatment effects on secondary forests were all insignificant.

**Table 3:** The effect of treatments on land cover (h/plot).

	Land cover outcomes	
	Δ Primary forest	Δ Secondary forest
***T1 (command-and-control measures) versus control***		
Mahalanobis w/2NN (M2NN)^a^	0.059 (0.072)	0.0132 (0.035)
Rosenbaum test (Γ)	-	-
**Marginal effect**^b^	**0.113 (0.069)**	**–0.0095 (0.021)**
Kernel PS (K-PS)^a^	0.107** (0.05)	–0.005 (0.089)
Rosenbaum test (Γ)	2.7	-
**Marginal effect**^b^	0.142** (0.0664)	**–0.0245 (0.027)**
Radius PS (R-PS)^a^	0.085* (0.048)	–0.005 (0.086)
Rosenbaum test (Γ)	1.5	-
**Marginal effect**^b^	0.112** (0.0533)	**-0.0199 (0.0266)**
***T2 (PES–ICDP mix) vs control***		
Mahalanobis w/2NN (M2NN)^a^	0.083**(0.038)	–0.076 (0.062)
Rosenbaum test (Γ)	2.1	-
**Marginal effect**^b^	0.134** (0.056)	**–0.075 (0.056)**
Kernel PS (K-PS)^a^	0.182*** (0.04)	–0.105 (0.105)
Rosenbaum test (Γ)	8.5	-
**Marginal effect**^b^	0.204*** (0.0682)	–0.121* (0.0691)
Radius PS (R-PS)^a^	0.189*** (0.04)	–0.115 (0.105)
Rosenbaum test (Γ)	8.9	-
**Marginal effect**^b^	0.199*** (0.0675)	–0.120* (0.069)

Note:

^(a)^ Post-matching mean comparisons with caliper (0.05).

^(b)^ Weighted linear regressions run in the matched sample, using the same covariates as regressors to obtain bias-adjusted marginal effects.

Significance levels: 10%*, 5%** and 1%***.

Treatment 2 (PES–ICDP mix added to command and control) significantly amplified the mitigating effect on primary forest loss; on average, it contributed to conserving somewhere between 0.13 ha (M2NN) and 0.2 ha (K-PS) of primary forest that otherwise would have been lost during the period from 2010 to 2016. All marginal treatment effects were larger and gained significance (1–5%) compared to T1. However, the K-PS and R-PS results indicated that secondary forest loss may also have risen slightly, yet the parameters were only significant at the 10% level. In conclusion, we reconfirm small but significant conservation effects from command-and-control measures (T1) on primary forests, which are further incrementally increased from the PES–ICDP mix treatment (T2).

### Effects on household wellbeing

Next, we estimated the effect of each treatment on household wellbeing indicators for the same period (2010–2016) (see Table 4). For household incomes, PS-K and PS-R estimates for the command-and-control treatment (T1) were, as expected, negative but insignificant; for M2NN they were positive. All asset effects of T1 were insignificant. T1-treated households reported significantly less improvement in their quality of life than controls; the estimates’ significance varies from 1% to 5% across matching methods.

**Table 4:** The effects of treatments on household wellbeing.

	OUTCOMES			
	Δ Income (PEN per year)	Δ Assets index	Perceived wellbeing	
			Improved	Worsening
***T1 (command-and-control measures) versus control***				
Mahalanobis w/2NN (M2NN)^a^	806.39* (450.82)	–0.45 (0.46)	–0.25** (0.126)	0.05 (0.0846)
Rosenbaum test (Γ)	1.5	-	2.0	-
**Marginal effect**^b^	593.01* (348.47)	**–0.45 (0.59)**	–0.28*** (0.11)	**–0.018 (0.03)**
Kernel PS (K-PS)^a^	–68.64 (901.57)	0.297 (0.569)	–0.283 (0.24)	–0.008 (0.109)
Rosenbaum test (Γ)	-	-	-	-
**Marginal effect**^b^	**–52.30 (297.66)**	**0.18 (0.284)**	–0.35** (0.155)	**0.00 (0.00)**
Radius PS (R-PS)^a^	–106.76 (564.80)	0.141 (0.577)	–0.208 (0.173)	–0.011 (0.088)
Rosenbaum test (Γ)	-	-	-	-
**Marginal effect**^b^	**–25.18 (374.52)**	**0.441 (0.277)**	–0.26* (0.136)	**0.00 (0.00)**
***T2 (PES–ICDP mix) versus control***				
Mahalanobis w/2NN (M2NN)^a^	810.77* (460.97)	0.393 (0.402)	–0.417*** (0.125)	0.21** (0.093)
Rosenbaum test (Γ)	1.0	-	3.5	1.7
**Marginal effect**^b^	**827.38 (508.1)**	**0.362 (0.557)**	–0.50*** (0.175)	**0.117 (0.086)**
Kernel PS (K-PS)^a^	1184.0 (801.98)	0.81 (0.496)	–0.498*** (0.157)	0.19 (0.124)
Rosenbaum test (Γ)	-	-	2.9	-
**Marginal effect**^b^	1021.5** (475.18)	0.757** (0.36)	–0.472*** (0.151)	0.132** (0.07)
Radius PS (R-PS)^a^	1121.36 (802.65)	0.814 (0.496)	–0.481*** (0.157)	0.193 (0.124)
Rosenbaum test (Γ)	-	-	2.9	-
**Marginal effect**^b^	988.99** (483.84)	0.761** (0.349)	–0.445*** (0.157)	0.125* (0.06)

Note:

^(a)^ Post-matching mean comparisons with caliper (0.05).

^(b)^ Weighted linear (for income and assets) and logit (for both perceived wellbeing indicators) regressions were run with the matched sample to obtain bias-adjusted marginal effects. We used the treatment and same covariates as regressors.

Significance levels: 10%*, 5%** and 1%***.

Regarding T2 (PES–ICDP mix), we found evidence of significantly positive effects on both income and assets (5% significance for K-PS and R-PS). The annual household incomes were PEN989–1022 (USD300–310) higher in this subgroup compared to controls, and asset values grew by 0.8 index points more than controls, using the estimated marginal effects after K-PS and R-PS matching (presenting best covariate balances). Conversely, there was a strongly negative impact on households’ perceived wellbeing: incentive-receiving households were statistically significantly (1% level) less likely to state that their wellbeing had improved by a probability ranging from 44% to 50% (using K-PS and R-PS algorithms).

## Discussion and concluding remarks

### Land cover effects

In Moyobamba, a set of interventions to protect strategic micro-watersheds for municipal water supply has been undertaken over the last decade, with the aim of reducing erosive sedimentation and enhancing dry season flows. Designed as a complex hybrid between PES, ICDP, protected sustainable use areas and conditional land rights, the initiative aimed to halt deforestation and promote sustainable agriculture, including agroforestry, while also improving livelihoods for upstream farmers. Sizeable investments were made, principally through various waves of sustainable development projects heavily subsidised by external donors, given that water user fees were never enough to cover the costs.

We now investigate the results emerging from the impact evaluation above (see Table 3). Splitting the complex intervention mix analytically into a ‘sticks only’ (T1: protected-area establishment, implicitly including conditional land rights) and ‘sticks and carrots’ treatment (T2: PA and PES-like conditional ICDP incentives), we find that the two treatments, separately and jointly, mitigated primary forest clearing in the expected reinforcing way. There were approximately equal quantitative contributions from both treatment components, but for T1, only the two propensity score methods yielded significant positive estimates (0–0.13 ha), while for T2 (0.13–0.20 ha), all three methods demonstrated a significant positive effect.

However, the evaluated land cover impacts of the initiative were, in absolute terms, quite small. We can calculate impacts as ranges obtained from the marginal parameters in the T1 and T2 sample quantifications and then scale them up to the entire population that was affected by the two treatments. From T1, multiplying the K-PS and R-PS estimators with the number of sampled plots provides a range of 2.91 ha to 3.83 ha. Assuming these estimates are extrapolable to the entire number of plots in the ZOCRE that did not receive rewards would yield a range of 10.04 ha to 13.22 ha of additional primary forest. This represents the estimated number of hectares that would counterfactually have been deforested, absent the declaration of the ZOCRE (T1). Similarly, for T2, the in-sample effect is 6.97 ha to 7.96 ha. This, upscaled to the total number of contractees, yields 7.91 ha to 9.03 ha that additionally would have been deforested by PES recipients, had they counterfactually not received any rewards. Hence, the combined, accumulated and upscaled impact of the two interventions over six years (2010–2016) was in the range of 7.91 ha to 22.25 ha of primary forest saved.

In response to this, we ask: why are we finding these arguably low land cover impacts from the implemented initiative? A partial answer is that the deforestation pressure in the entire area was not too large from the outset of the intervention. This seems at least in part related to the outbreak of the coffee plant plague known locally as *la roya amarilla* (*Puccinia striiformis*), which hit the region particularly between 2011 and 2016, thereby greatly reducing the attractiveness of converting forest to coffee plantations—previously the prime driver of deforestation [44].

A second internal, though minor, reason is that the initiative did not manage to entirely stop deforestation in the target zone, as might have been expected from the joint application of the ZOCRE and PES contract rules. This has to do with the monitoring and sanctioning mechanisms, together making up the principle of (enforced) conditionality. This is frequently referred to as the principal innovation of PES schemes [65, 66] but, in practice, often proves ill enforced [67]. While a protocol to monitor conservation agreements was established locally [68], few of the indicators were defined at plot level (i.e., at a sufficiently fine scale to capture potential violations). Notably, some clearings of secondary forest were, at pressure from farmers, also being allowed to establish ‘eco-friendly agricultural practices’ such as coffee agroforestry systems. However, posterior adoption was not properly ground-monitored and more traditional coffee farming was brought in, rather than agroforestry practices (e.g., use of shadow trees, fences). The official monitoring guide of the initiative [68] relies as protocol on self-reported information from interviews, rather than independent verification. The progress reports we reviewed during fieldwork also mainly monitored the delivery of benefits to signatory households, rather than their environmental compliance.

In prolongation, benefit delivery was also not consistently conditional upon land use compliance. Interviews with various stakeholders revealed that some in-kind rewards, such as seedlings or training, were not delivered exclusively to signatory households but instead to all ZOCRE-settled households, to give the initiative greater legitimacy. Furthermore, the payments were also discontinued; the last conservation agreements expired in 2015 due to shortage of funds. While some households still maintain hopes for further rounds of benefits, their patience has been tested over the last few years. Obviously, they likely still benefit from past ICDP investments in apiculture, handicraft, tourism and shade-grown coffee production.

Regarding sanctions, the initiative does not belong to those types of PES schemes that never punished any noncompliance—not only has it been known to cut off future incentives, but it has also enacted retroactive punishment. We collected testimonies of at least one emblematic case of a PES contract signatory who went to jail, having grossly violated the forest clearing rules on his plot. Therefore, problem of conditionality in Moyobamba seems more related to imprecise environmental monitoring at plot level than to detected but unsanctioned noncompliance.

### Livelihood effects

Our results show that households receiving sticks only treatment (T1) suffered, as expected, some setbacks in self-perceived livelihood indicators, while income and asset effects were mostly insignificant. Unsurprisingly, households receiving both sticks and carrots (T2) were well compensated, and registered, on average, an income increase eight times larger than the controls (naïvely compared in Table 2). Although variance was large, a significant increase in annual incomes (USD300–310 per household) and assets persisted after matching. There are good reasons for this. As documented in the project timeline (see Fig 1), several cumulative waves of sizeable project investments favoured the relatively small group of PES-contracted households. The seeming paradox is, therefore, that the same group of T2 households that celebrated significant advances in material welfare exhibited a strongly negative self-perception of how their wellbeing developed over the same time period (see Table 4). In part, this probably has to do with perception of increased land tenure insecurity, which affects the entire population in the ZOCRE sustainable-use area (see the section dedicated to land rights below); both groups were less likely to state improvements in self-perceived wellbeing than control households.

However, for T1, the size and significance of this effect was not as marked and consistent over matching methods as for T2. Moreover, T2 households were also significantly more likely to state outright decreases in wellbeing than controls; this was not the case for T1. This raises the question: why is the most income- and asset-expanding group of households, the amply incentive-treated T2, clearly voicing the greatest dissatisfaction?

We suggest that this likely relates to the fact that large incentives were given and so became embodied into beneficiaries’ future expectations, but then benefit flows were discontinued in 2015. For a minimum of three years (for some, considerably more), PES contractees have become frustrated by not receiving any (new) payments or rewards, nor having clear prospects for benefit flows to be reinitiated any time soon. It is common, especially in ICDPs that tend to create paternalistic expectations on both sides, that beneficiaries voice strong dissatisfaction with spending-intensive projects when these, for whatever reason, have to cut back on their investments [48]. The Noel Kempff project in Bolivia is one such prominent example in Latin America [69]. This negative expression of trends in wellbeing should likely be understood predominantly as a vote of protest.

### Land rights

A distinct feature of the Moyobamba initiative is that settlers in the protected sustainable-use area (ZOCRE) hold land rights that are conditional upon their environmentally benign stewardship; settlers could be expelled or put into prison in the case of blatant legal noncompliance, and at least one such demonstrative case exists (see section dedicated to land cover effects). Notably, local obligations also include communally organised monitoring vis-a-vis prospective land occupation from outside [70]. The case resembles others in the PES literature in which withdrawable land rights are being used as part of environmental conditionality (e.g., [66, 71]), though only a few Asian cases were specifically being formally reported (e.g., in Indonesia [72] and Cambodia [39]).

Currently, the regional government has announced plans to outsource management of the ZOCRE (e.g., to an environmental non-governmental organisation or another government agency), citing the high costs of self-administration as its main motive. Our interviews revealed that the resulting insecurity about future rules and conditions heavily influenced the negative household self-perceived wellbeing responses received from ZOCRE residents, especially as this decision seemingly was made without local consultation [70].

### Cost-efficiency

As noted in the project timeline above (see Fig 1), sizeable investments have been made in the project. Using the information available at the *Pre Registro Nacional de Mecanismos de Servicios Ecosistémicos* [73], we can estimate the per-area amount invested in the frame of the Moyobamba initiative and compare it with other national experiences that present similar advances in their implementation. We find that Moyobamba is the single most spending-intense initiative. In total, USD353/ha have been spent so far, compared to an average of USD17/ha among four other similar, though admittedly shorter-lived, initiatives (in Cumbaza, Chira, Tilachancha and Quanda). In times when PESs were generally considered novel and promising, Moyobamba became a Peruvian ‘donor darling’ that was an attractive investment in terms of piloting watershed management incentives.

We can compare the scheme’s spending intensity not only with respect to the area enrolled, but also regarding its environmental additionality—that is, the number of hectares of forests that the initiative has saved from clearing, according to our above calculations. Combining the spending numbers previously mentioned (for the incentive component) with the impact ranges discussed above, we find that the initiative, with its cumulative investments, has had a high cost of USD24,900/ha–USD70,041/ha of forest saved. Given the impossibility of separating T1 and T2 costs, we divided the inflation-adjusted total updated costs of the initiative—USD554,024 (S3 Table)—by our estimated primary forest saved by T1 plus T2, estimated in the range between 7.91 ha and 22.25 ha. This amount is equivalent to an average annual cost between USD2075/ha and USD5837/ha for the period from 2004 to 2016.

A second bottom line of the project relates to human wellbeing impacts. Table 4 illustrates that T2 (PES–ICDP mix) caused incremental household annual incomes between USD300 and USD310 per household. Dividing the total spending of the project on the incentive component (PEN224,963/USD68,171 [74]) by the 42 sampled households treated by T2, the average project incentive spending (T2) per contractually participating household was USD1623—more than five times the average rise in 2016 household annual income. It remains to be observed to what extent the sunk ICDP investments will continue to yield extra household incomes in the future, now that technical assistance has been reduced. These results also lead to speculation regarding the extent to which ICDP investments were an efficient vehicle in terms of welfare, or whether direct cash payments to households could potentially have boosted livelihoods more efficiently.

These results paint a somewhat sobering picture about the cost-effectiveness of the initiative; each hectare saved and *Soles* (PEN) of incremental household income created came at high cost. Some unpredictable factors influenced environmental outcomes, such as the coffee disease–led downturn in local ‘baseline’ deforestation pressures, resulting in the low measured additionality. Overall, Moyobamba was the first ever PES project in Peru; pilots are to be experimental in nature and are seldom particularly cost-efficient. By our interpretation, the project should be given ample margin.

### Perspectives and policy recommendations

Recently, direct conservation incentives have been considered promising ways to deal with threats to ecosystem services, including in watersheds [75, 76]. PES, the most emblematic of these policies, should bring about environmental outcomes in a more effective and cost-efficient way [19, 77, 78], while simultaneously alleviating poverty [21, 79].

Moyobamba is Peru’s pioneer and flagship PES case, used for replication in other watersheds [17]. A comparable PES-cum-ICDP intervention mix has also been applied by the Ministry of Environment’s National Programme for Forest Conservation (PNCB), which in Amazonian communities is using conditional incentives combined with investments in productive alternatives to promote forest conservation [11]; however, it is reportedly yielding equally modest environmental outcomes [12]. Since it is the most influential PES pilot in Peru, it is important to understand the Moyobamba project’s impacts to date, drawing adequate lessons for similar future initiatives.

First, we suggest that performance might have been better had the initiative been allowed invest more in the PES component and less in the ICDP component. As demonstrated, the ICDP investments proved to have little cost-efficiency regarding both environmental and income-generating goals. In retrospect, the optimistic pre-analysis of productive alternatives (e.g., [44]) perhaps underestimated the costs, obstacles and (plant disease) risks of a transition to perennial agroforestry. Project investments proved too ambitious to be covered by water user fees throughout, relying instead on punctual donor hyper-injections that raised the bar of beneficiary expectations but were eventually disrupted, contributing to the negative wellbeing perceptions among service providers.

Second, and relatedly, the initiative did not monitor compliance reliably at the farm scale and may have allowed for some secondary forest clearing for the establishment of (supposedly) shade-grown coffee systems. This feature compromised conditionality and is critical to many PES initiatives [67, 80]. Eventually, slack conditionality was not in the interest of focused ecosystem service provision, thereby also jeopardising environmental performance. Nevertheless, the conditional land tenure element seemed to have worked well across the ZOCRE as an alternative element of environmental conditionality; however, as a regulatory ‘stick’, it also negatively affected land users’ perceptions of wellbeing.

In this respect, Moyobamba exhibits some commonalities with other PES-like initiatives in the Amazon, such as Brazil’s Proambiente program [81], Bolsa Floresta in Brazil’s Amazonas state [82], the Transamazon project in Pará, also in Brazil [59], Peru’s aforementioned national PNCB program [11, 12] and Ecuador’s Socio Bosque [83]. For a variety of reasons, all implementers placed significantly more faith in the ICDP-type model of directed productive change—of allegedly ‘solving the problem’ permanently—than in the power of conditional payments. In these schemes, PES became part of highly complex intervention mixes, with other components sometimes having larger attributive roles in shaping on-the-ground impacts. Needless to say, these complex implementation designs also limit the options for evaluating the extent to which PES was genuinely working towards the intended environmental and livelihoods objectives.

Finally, we have only examined land cover proxy effects, not hydrological service end delivery, which sometimes might lead to erroneous conclusions [84, 85]. In Moyobamba, detailed socio-hydrological pre-analysis based on soil and water assessment tool models had neatly identified eight hydrological response units within the Mishquiyacu micro-watershed as being highly critical to erosion costs of the EPS Moyobamba [44]. However, the eventual implementation of the rewards mechanism was much less spatially targeted and, perhaps, more aligned with a broad development project logic whereby, typically, from an equity perspective, nobody should be left out.

Given this evaluation, we make the following recommendations regarding the Moyobamba initiative. More modest, continuous and truly conditional payments, aligned closer with the (equally continuous) water user payments, and less donor spree–dependent investments, may provide for a more sustainable future pathway. If cash payments to landowners remain politically infeasible, in-kind transfers for current consumption could still be an alternative, rather than forced investment projects resulting in high transaction costs, risks and expectations. If budgets are tight, highest spatial priority should go to payments in the pre-identified, most erosion-prone areas, which leverage by far the highest environmental service. Reducing add-on ICDP features and moving the Moyobamba initiative more towards a proper, spatially well-targeted PES intervention might eventually improve both its environmental and livelihoods impacts.

## Supporting information

S1 Table Relevant impacts evaluation of PES. (DOCX)

S2 Table Covariate balance for land-cover and wellbeing analysis. (DOCX)

S3 Table Additional estimations. (DOCX)

S1 Dataset Stata archives (dta and do files) required to replicate findings reported in this paper are available as supporting information file. (ZIP)
