# Supplementary material for: Adding rewards to regulation: The impacts of watershed conservation on land cover and household wellbeing in Moyobamba, Peru
Source: PLoS One. 2019 Nov 20;14(11):e0225367. doi: 10.1371/journal.pone.0225367 (PMC6867640; doi:10.1371/journal.pone.0225367)
Supplement: S1 Table — (DOCX) [file pone.0225367.s001.docx]

**S1 Table. Relevant impacts evaluation of PES.**

| **Study & PES name^a^** | **Unit of analysis** | **Period** | **Total sample** | **Methods** | **Results** |
| --- | --- | --- | --- | --- | --- |
| Robalino & Pffaf [23]  Pagos por Servicios Ambientales,  Costa Rica | Pixel | 1997-2000 | 10.106 (6% treated; 94% controls) | Matching and adjustment regression | Deforestation reduced by 1%. |
| Arriagada, et al. [24]  Pagos por Servicios Ambientales, Costa Rica (Sarapiquí) | Farm | 1996-2005 | 50 treated  152 controls | Matching and adjustment regression | PES contribute to conserve additional 11 to 17% of forest cover in enrolled farms. |
| Arriagada, et al. [25]  Pagos por Servicios Ambientales, Costa Rica (Sarapiquí) | Household | 1996-2005 | 50 treated  152 controls | Matching and adjustment regression | No significant PES effects on participants’ welfare. |
| Alix-García, et al. [27]  Payment for Hydrological Ecosystem Services, Mexico | Parcel (250x250 m pixels) | 2003-2006 | 352 treated  462 control | Matching and Tobit regression | Deforestation reduced by 50% (but low absolute impact). |
| Alix-García, et al. [28]  Payment for Hydrological Ecosystem Services, Mexico | *Environmental analysis:*  Fixed points  *Socioeconomic analysis:*  Households | *Environmental analysis:*  2003-2011  *Socioeconomic analysis:*  2000-2010 | *Envir. analysis:*  17,307 treated, and 18,456 controls  *Socioec. analysis:*  590 treated, and 506 non-treated | Matching and fixed effect regression | The scheme reduced the expected land cover loss by [40–51]% and generates small but positive poverty alleviation. |
| Costeadoat, et al. [29]  Payment for Hydrological Ecosystem Services, Mexico (Chiapas) | Grid cell (10 Ha) | 2007-2013 | 1413 treated, 761 controls | Matching and fixed effect regression | Deforestation reduced by 12-14.7%. |
| Sims and Alix-García [30]  México | Localities (Mexican census unit) | Land cover changes: 2000-2012  Poverty: 2000-2010 | 4984 treated, 59,551 control | Matching and fixed effects regressions | Deforestation cut by 24%, poverty by 12% (compared to projection). |
| Le Velly, et al. [31]  Payment for Hydrological Ecosystem Services, Mexico (Yucatán) | Polygons | 2005-2012 | 4,963 treated and 5,389 untreated | Propensity score matching and OLS | Deforestation is reduced by 2.45% on enrolled parcels. |
| Arriagada, et al. [32]  Payment for Hydrological Ecosystem Services, Mexico | Households | 2007-2013 | 610 treated and 1550 untreated | Matching and lineal regression | No differences in conventional wellbeing indicators but it is alleged that participant households significantly increased their (subjective) valuation over forests ecosystems services. |
| Uchida, et al. [33]  Grain-for-Green, China | Household | 1999-2004 | 270 households entered PES programme sequentially (maintaining control groups per year) | Matching and fixed effect regression | PES enrolled adults increased their participation in off-farm jobs by 15-20%. |
| Fu, et al. [34]  Grain-for-Green, China | Counties | 1998-2008 | 252 treated and 157 non-treated | Propensity Score Matching and fixed effect regression | The program has contributed to conserve 621 ha of forests in PES prioritized counties. |
| Jones, et al. [35]  Socio Bosque  Ecuador (Cuyabeno) | Parcel | 2011-2013 | 63 treated,  450 controls | Matching and fixed effect regression | Annual deforestation reduced by 0.4-0.5% (two thirds of deforestation risk). |
| Cuenca, et al. [36]  Socio Bosque  Ecuador | Cells | 2008-2014 | 30 439 treated, 260 050 controls | Propensity score matching and lineal regression | Annual deforestation reduced by 1.5% |
| Giudice, et al. [12]  Programa Nacional de Conservación de Bosques, Peru | Communities | 2011-2015 | 50 treated and 36 untreated | Matching and fixed effects regression | The program has contributed to conserve 557 ha for the analysed period corresponding to a 5.8% deforestation reduction. |
| Zheng, et al. [38]  Paddy Land-to-Dry Land  Miyun Reservoir Watershed, China | Household | 2006-2010 | 394 participants, 329 non participants | Matching and fixed effects regression | Resident agricultural incomes decreased; migrant income increased. |
| Clements and Milner-Gulland [39]  Two PA in Northern Cambodia | *Deforestation:*  1-km grid square  *Welbeing:*  Household | 2005-2010 | *Defor.:* 1356 treated, 1948 controls.  *Welb.:* 443 treated, 326 controls | *Defor.:*  Matching  *Welb.:*  Matching and regression | PES in PA reduced deforestation by 50% and increased wellbeing by 6% |
| Jones, et al. [40]  Decentralized Payment for Hydrological Services (PHS) program  Chiapas, Mexico | Households | 2010-2015 | Treated: 62, non-treated: 70. | Matching and regression | Participant households increased their assets in 0.5 asset index point. |

(^a^) Number in brackets correspond to the source.
