# Supplementary material for: Adding rewards to regulation: The impacts of watershed conservation on land cover and household wellbeing in Moyobamba, Peru
Source: PLoS One. 2019 Nov 20;14(11):e0225367. doi: 10.1371/journal.pone.0225367 (PMC6867640; doi:10.1371/journal.pone.0225367)
Supplement: S2 Table — (DOCX) [file pone.0225367.s002.docx]

**S2 Table. Covariate balance for land-cover and wellbeing analysis.**

**S2A Table. Covariate balance achieved to compare T1 (*command-and-control* measures) vs control plots groups using the M2NN matching algorithm.**

| **Covariate** | **Unmatched (U)/ Matched (M)** | **Mean** | | **% St. Dif.** | **t-test** | |
| --- | --- | --- | --- | --- | --- | --- |
|  |  | **Treated** | **Control** |  | **t** | **p > \|t\|** |
| Plot size (ha) | U | 5.4469 | 2.4504 | 59.9 | 4.16 | 0.000 |
|  | M | 5.4469 | 3.0347 | 48.2 | 2.31 | 0.023 |
| Slope (degrees) | U | 16.093 | 4.2138 | 241.6 | 18.09 | 0.000 |
|  | M | 16.093 | 12.49 | 73.3 | 3.07 | 0.003 |
| Altitude (m) | U | 980.98 | 867.84 | 133.2 | 12.39 | 0.000 |
|  | M | 980.98 | 938.05 | 50.6 | 2.24 | 0.027 |
| Distance to Moyobamba (km) | U | 9.64 | 8.3026 | 61.7 | 3.01 | 0.003 |
|  | M | 9.64 | 9.7894 | -6.9 | -0.45 | 0.653 |
| Forest cover 2010 (Ha) | U | 2.2752 | .6027 | 76.1 | 6.06 | 0.000 |
|  | M | 2.2752 | 1.0329 | 56.5 | 2.62 | 0.011 |
| Δ Primary forest 2005-2010 (ha/plot) | U | -.16815 | -.03711 | -42.4 | -3.95 | 0.000 |
|  | M | -.16815 | -.11114 | -18.4 | -0.71 | 0.480 |
| Secondary forest 2010 (Ha) | U | 2.4167 | .92582 | 59.5 | 5.02 | 0.000 |
|  | M | 2.4167 | 1.3218 | 43.7 | 1.86 | 0.066 |
| Δ Secondary forest 2005-2010 (ha/plot) | U | .56674 | .10593 | 42.6 | 3.27 | 0.001 |
|  | M | .56674 | .27974 | 26.5 | 1.16 | 0.249 |

**S2B Table. Covariate balance achieved to compare T2 (*PES-ICDP* mix) vs control plots groups using the M2NN matching algorithm.**

| **Covariate** | **Unmatched (U)/ Matched (M)** | **Mean** | | **% St. Dif.** | **t-test** | |
| --- | --- | --- | --- | --- | --- | --- |
|  |  | **Treated** | **Control** |  | **t** | **p > \|t\|** |
| Plot size (ha) | U | 4.2824 | 2.4504 | 43.0 | 2.87 | 0.004 |
|  | M | 4.2824 | 2.7354 | 36.3 | 2.13 | 0.036 |
| Slope (degrees) | U | 14.32 | 4.2138 | 219.8 | 16.96 | 0.000 |
|  | M | 14.32 | 11.223 | 67.4 | 3.11 | 0.002 |
| Altitude (m) | U | 992.1 | 867.84 | 135.0 | 13.84 | 0.000 |
|  | M | 992.1 | 943.25 | 53.1 | 2.53 | 0.013 |
| Distance to Moyobamba (km) | U | 8.2696 | 8.3026 | -0.8 | -0.07 | 0.945 |
|  | M | 8.2696 | 7.6777 | 14.3 | 0.70 | 0.488 |
| Forest cover 2010 (Ha) | U | 1.8964 | .6027 | 52.1 | 4.80 | 0.000 |
|  | M | 1.8964 | 1.1345 | 30.7 | 1.54 | 0.127 |
| Δ Primary forest 2005-2010 (ha/plot) | U | -.06768 | -.03711 | -16.4 | -1.18 | 0.239 |
|  | M | -.06768 | -.02493 | -22.9 | -1.21 | 0.228 |
| Secondary forest 2010 (Ha) | U | 1.8602 | .92582 | 60.1 | 3.89 | 0.000 |
|  | M | 1.8602 | .95182 | 58.5 | 3.42 | 0.001 |
| Δ Secondary forest 2005-2010 (ha/plot) | U | .57672 | .10593 | 47.0 | 3.69 | 0.000 |
|  | M | .57672 | .17678 | 39.9 | 1.89 | 0.062 |

**S2C Table. Covariate balance achieved to compare T1 (*command-and-control* measures) vs control plots groups using the K-PS matching algorithm.**

| **Covariate** | **Unmatched (U)/ Matched (M)** | **Mean** | | **% St. Dif.** | **t-test** | |
| --- | --- | --- | --- | --- | --- | --- |
|  |  | **Treated** | **Control** |  | **t** | **p > \|t\|** |
| Plot size (ha) | U | 5.4469 | 2.4504 | 59.9 | 4.16 | 0.000 |
|  | M | 5.0808 | 3.0153 | 41.3 | 1.51 | 0.138 |
| Slope (degrees) | U | 16.093 | 4.2138 | 241.6 | 18.09 | 0.000 |
|  | M | 13.298 | 13.588 | -5.9 | -0.23 | 0.819 |
| Altitude (m) | U | 980.98 | 867.84 | 133.2 | 12.39 | 0.000 |
|  | M | 932.81 | 953.93 | -24.9 | -1.01 | 0.316 |
| Distance to Moyobamba (km) | U | 9.64 | 8.3026 | 61.7 | 3.01 | 0.003 |
|  | M | 9.5959 | 9.7101 | -5.3 | -0.25 | 0.807 |
| Forest cover 2010 (Ha) | U | 2.2752 | .6027 | 76.1 | 6.06 | 0.000 |
|  | M | 1.9067 | 1.0316 | 39.8 | 1.46 | 0.151 |
| Δ Primary forest 2005-2010 (ha/plot) | U | -.16815 | -.03711 | -42.4 | -3.95 | 0.000 |
|  | M | -.20666 | -.11176 | -30.7 | -0.85 | 0.399 |
| Secondary forest 2010 (Ha) | U | 2.4167 | .92582 | 59.5 | 5.02 | 0.000 |
|  | M | 2.1264 | 1.4158 | 28.3 | 1.01 | 0.319 |
| Δ Secondary forest 2005-2010 (ha/plot) | U | .56674 | .10593 | 42.6 | 3.27 | 0.001 |
|  | M | .30849 | .25674 | 4.8 | 0.19 | 0.847 |

**S2D Table. Covariate balance achieved to compare T2 (*PES-ICDP* mix) vs control plots groups using the K-PS matching algorithm.**

| **Covariate** | **Unmatched (U)/ Matched (M)** | **Mean** | | **% St. Dif.** | **t-test** | |
| --- | --- | --- | --- | --- | --- | --- |
|  |  | **Treated** | **Control** |  | **t** | **p > \|t\|** |
| Plot size (ha) | U | 4.2824 | 2.4504 | 43.0 | 2.87 | 0.004 |
|  | M | 3.9144 | 3.9278 | -0.3 | -0.01 | 0.991 |
| Slope (degrees) | U | 14.32 | 4.2138 | 219.8 | 16.96 | 0.000 |
|  | M | 12.581 | 12.307 | 6.0 | 0.24 | 0.810 |
| Altitude (m) | U | 992.1 | 867.84 | 135.0 | 13.84 | 0.000 |
|  | M | 953.74 | 947.16 | 7.1 | 0.35 | 0.724 |
| Distance to Moyobamba (km) | U | 8.2696 | 8.3026 | -0.8 | -0.07 | 0.945 |
|  | M | 7.6945 | 8.4051 | 17.2 | -0.83 | 0.408 |
| Forest cover 2010 (Ha) | U | 1.8964 | .6027 | 52.1 | 4.80 | 0.000 |
|  | M | 1.561 | 1.4628 | 4.0 | 0.16 | 0.874 |
| Δ Primary forest 2005-2010 (ha/plot) | U | -.06768 | -.03711 | -16.4 | -1.18 | 0.239 |
|  | M | -.06417 | -.04304 | -11.3 | -0.49 | 0.628 |
| Secondary forest 2010 (Ha) | U | 1.8602 | .92582 | 60.1 | 3.89 | 0.000 |
|  | M | 1.7222 | 1.716 | 0.4 | 0.01 | 0.990 |
| Δ Secondary forest 2005-2010 (ha/plot) | U | .57672 | .10593 | 47.0 | 3.69 | 0.000 |
|  | M | .59931 | .43704 | 16.2 | 0.56 | 0.577 |

**S2E Table. Covariate balance achieved to compare T1 (*command-and-control* measures) vs control plots groups using the R-PS matching algorithm.**

| **Covariate** | **Unmatched (U)/ Matched (M)** | **Mean** | | **% St. Dif.** | **t-test** | |
| --- | --- | --- | --- | --- | --- | --- |
|  |  | **Treated** | **Control** |  | **t** | **p > \|t\|** |
| Plot size (ha) | U | 5.4469 | 2.4504 | 59.9 | 4.16 | 0.000 |
|  | M | 4.9389 | 3.0524 | 37.7 | 1.33 | 0.189 |
| Slope (degrees) | U | 16.093 | 4.2138 | 241.6 | 18.09 | 0.000 |
|  | M | 13.209 | 13.452 | -4.9 | -0.19 | 0.853 |
| Altitude (m) | U | 980.98 | 867.84 | 133.2 | 12.39 | 0.000 |
|  | M | 929.35 | 952.99 | -27.8 | -1.11 | 0.272 |
| Distance to Moyobamba (km) | U | 9.64 | 8.3026 | 61.7 | 3.01 | 0.003 |
|  | M | 9.6335 | 9.6377 | -0.2 | -0.01 | 0.993 |
| Forest cover 2010 (Ha) | U | 2.2752 | .6027 | 76.1 | 6.06 | 0.000 |
|  | M | 1.6969 | 1.0076 | -31.4 | 1.19 | 0.241 |
| Δ Primary forest 2005-2010 (ha/plot) | U | -.16815 | -.03711 | -42.4 | -3.95 | 0.000 |
|  | M | -.19804 | -.10817 | 29.0 | -0.78 | 0.438 |
| Secondary forest 2010 (Ha) | U | 2.4167 | .92582 | 59.5 | 5.02 | 0.000 |
|  | M | 2.1529 | 1.455 | 27.9 | 0.95 | 0.347 |
| Δ Secondary forest 2005-2010 (ha/plot) | U | .56674 | .10593 | 42.6 | 3.27 | 0.001 |
|  | M | .29443 | .27887 | 1.4 | 0.06 | 0.995 |

**S2F Table. Covariate balance achieved to compare T2 (*PES-ICDP* mix) vs control plots groups using the R-PS matching algorithm.**

| **Covariate** | **Unmatched (U)/ Matched (M)** | **Mean** | | **% St. Dif.** | **t-test** | |
| --- | --- | --- | --- | --- | --- | --- |
|  |  | **Treated** | **Control** |  | **t** | **p > \|t\|** |
| Plot size (ha) | U | 4.2824 | 2.4504 | 43.0 | 2.87 | 0.004 |
|  | M | 3.9144 | 4.1693 | -6.0 | -0.19 | 0.848 |
| Slope (degrees) | U | 14.32 | 4.2138 | 219.8 | 16.96 | 0.000 |
|  | M | 12.581 | 12.285 | 6.4 | 0.26 | 0.797 |
| Altitude (m) | U | 992.1 | 867.84 | 135.0 | 13.84 | 0.000 |
|  | M | 953.74 | 946.88 | 7.5 | 0.37 | 0.713 |
| Distance to Moyobamba (km) | U | 8.2696 | 8.3026 | -0.8 | -0.07 | 0.945 |
|  | M | 7.6945 | 8.2636 | -13.8 | -0.66 | 0.511 |
| Forest cover 2010 (Ha) | U | 1.8964 | .6027 | 52.1 | 4.80 | 0.000 |
|  | M | 1.561 | 1.5418 | 0.8 | 0.03 | 0.977 |
| Δ Primary forest 2005-2010 (ha/plot) | U | -.06768 | -.03711 | -16.4 | -1.18 | 0.239 |
|  | M | -.06417 | -.04649 | -9.5 | -0.39 | 0.700 |
| Secondary forest 2010 (Ha) | U | 1.8602 | .92582 | 60.1 | 3.89 | 0.000 |
|  | M | 1.7222 | 1.7745 | -3.4 | -0.11 | 0.915 |
| Δ Secondary forest 2005-2010 (ha/plot) | U | .57672 | .10593 | 47.0 | 3.69 | 0.000 |
|  | M | .59931 | .43831 | 16.1 | 0.54 | 0.589 |

**S2G Table. Covariate balance achieved to compare T1 (*command-and-control* measures) vs control households groups using the M2NN matching algorithm.**

| **Covariate** | **Unmatched (U)/ Matched (M)** | **Mean** | | **% St. Dif.** | **t-test** | |
| --- | --- | --- | --- | --- | --- | --- |
|  |  | **Treated** | **Control** |  | **t** | **p > \|t\|** |
| Total size of managed lands (ha) | U | 6.6865 | 4.1945 | 38.3 | 2.33 | 0.020 |
|  | M | 6.6865 | 4.0344 | 40.8 | 2.01 | 0.047 |
| Average slope (degrees) | U | 16.2 | 4.4367 | 238.8 | 17.00 | 0.000 |
|  | M | 16.2 | 11.9 | 87.3 | 3.61 | 0.001 |
| Average altitude (m) | U | 912.08 | 871.95 | 54.8 | 4.38 | 0.000 |
|  | M | 912.08 | 918.51 | -8.8 | -0.37 | 0.712 |
| Average distance to Moyobamba (km) | U | 9.738 | 7.9234 | 83.1 | 3.93 | 0.000 |
|  | M | 9.738 | 9.3156 | 19.3 | 1.37 | 0.174 |
| Total forest cover 2010 (ha/hh) | U | 5.8047 | 2.6165 | 63.3 | 4.51 | 0.000 |
|  | M | 5.8047 | 3.2148 | 51.4 | 2.25 | 0.027 |
| Δ Total forest 2005-2010 (ha/hh) | U | .48723 | .1178 | 29.4 | 2.00 | 0.046 |
|  | M | .48723 | .24925 | 19.0 | 0.90 | 0.371 |
| Household’ members 2010 | U | 3.075 | 2.9476 | 10.7 | 0.64 | 0.521 |
|  | M | 3.075 | 2.975 | 8.4 | 0.38 | 0.702 |
| Assets index 2010 | U | 2.625 | 3.5939 | -52.4 | -3.03 | 0.003 |
|  | M | 2.625 | 2.8625 | -12.8 | -0.63 | 0.529 |
| Income 2010 (PEN) | U | 8154.3 | 17664 | -66.2 | -3.13 | 0.002 |
|  | M | 8514.3 | 9418.1 | -6.5 | -0.49 | 0.623 |

**S2H Table. Covariate balance achieved to compare T2 (*PES-ICDP* mix) vs control households groups using the M2NN matching algorithm.**

| **Covariate** | **Unmatched (U)/ Matched (M)** | **Mean** | | **% St. Dif.** | **t-test** | |
| --- | --- | --- | --- | --- | --- | --- |
|  |  | **Treated** | **Control** |  | **t** | **p > \|t\|** |
| Total size of managed lands (ha) | U | 6.2444 | 4.1945 | 32.5 | 1.98 | 0.049 |
|  | M | 6.2444 | 3.977 | 35.9 | 1.89 | 0.062 |
| Average slope (degrees) | U | 14.476 | 4.4367 | 228.5 | 15.48 | 0.000 |
|  | M | 14.476 | 10.81 | 83.5 | 3.58 | 0.001 |
| Average altitude (m) | U | 936.21 | 871.95 | 80.8 | 6.77 | 0.000 |
|  | M | 936.21 | 922.05 | 17.8 | 0.76 | 0.448 |
| Average distance to Moyobamba (km) | U | 8.2455 | 7.9234 | 7.7 | 0.58 | 0.565 |
|  | M | 8.2455 | 7.8538 | 9.3 | 0.43 | 0.669 |
| Total forest cover 2010 (ha/hh) | U | 5.5891 | 2.6165 | 58.8 | 4.27 | 0.000 |
|  | M | 5.5891 | 3.1246 | 48.8 | 2.27 | 0.026 |
| Δ Total forest 2005-2010 (ha/hh) | U | .65257 | .1178 | 43.9 | 2.99 | 0.003 |
|  | M | .65257 | .25912 | 32.3 | 1.50 | 0.138 |
| Household’ members 2010 | U | 2.7381 | 2.9476 | -19.8 | -1.11 | 0.266 |
|  | M | 2.7381 | 2.8452 | -10.1 | -0.52 | 0.606 |
| Assets index 2010 | U | 2.4048 | 3.5939 | -56.9 | -3.64 | 0.000 |
|  | M | 2.4048 | 2.8333 | -20.5 | -0.97 | 0.335 |
| Income 2010 (PEN) | U | 9489.8 | 17664 | -55.5 | -2.83 | 0.005 |
|  | M | 9489.8 | 9165.4 | 2.2 | 0.17 | 0.869 |

**S2I Table. Covariate balance achieved to compare T1 (*command-and-control* measures) vs control households groups using the K-PS matching algorithm.**

| **Covariate** | **Unmatched (U)/ Matched (M)** | **Mean** | | **% St. Dif.** | **t-test** | |
| --- | --- | --- | --- | --- | --- | --- |
|  |  | **Treated** | **Control** |  | **t** | **p > \|t\|** |
| Total size of managed lands (ha) | U | 6.6865 | 4.1945 | 38.3 | 2.33 | 0.020 |
|  | M | 4.8414 | 4.3045 | 8.3 | 0.30 | 0.762 |
| Average slope (degrees) | U | 16.2 | 4.4367 | 238.8 | 17.00 | 0.000 |
|  | M | 13.053 | 12.888 | 3.3 | 0.11 | 0.916 |
| Average altitude (m) | U | 912.08 | 871.95 | 54.8 | 4.38 | 0.000 |
|  | M | 915.53 | 930.06 | -19.9 | -0.53 | 0.599 |
| Average distance to Moyobamba (km) | U | 9.738 | 7.9234 | 83.1 | 3.93 | 0.000 |
|  | M | 9.55 | 9.9777 | -19.6 | -1.02 | 0.317 |
| Total forest cover 2010 (ha/hh) | U | 5.8047 | 2.6165 | 63.3 | 4.51 | 0.000 |
|  | M | 3.8912 | 3.584 | 6.1 | 0.21 | 0.832 |
| Δ Total forest 2005-2010 (ha/hh) | U | .48723 | .1178 | 29.4 | 2.00 | 0.046 |
|  | M | .39754 | .5249 | -10.2 | -0.35 | 0.731 |
| Household’ members 2010 | U | 3.075 | 2.9476 | 10.7 | 0.64 | 0.521 |
|  | M | 3.2632 | 2.9969 | 22.4 | 0.63 | 0.533 |
| Assets index 2010 | U | 2.625 | 3.5939 | -52.4 | -3.03 | 0.003 |
|  | M | 2.9474 | 3.0319 | -4.6 | -0.17 | 0.868 |
| Income 2010 (PEN) | U | 8154.3 | 17664 | -66.2 | -3.13 | 0.002 |
|  | M | 8686.9 | 7755.3 | 6.7 | 0.35 | 0.727 |

**S2J Table. Covariate balance achieved to compare T2 (*PES-ICDP* mix) vs control households groups using the K-PS matching algorithm.**

| **Covariate** | **Unmatched (U)/ Matched (M)** | **Mean** | | **% St. Dif.** | **t-test** | |
| --- | --- | --- | --- | --- | --- | --- |
|  |  | **Treated** | **Control** |  | **t** | **p > \|t\|** |
| Total size of managed lands (ha) | U | 6.2444 | 4.1945 | 32.5 | 1.98 | 0.049 |
|  | M | 4.4736 | 3.4168 | 16.7 | 0.98 | 0.331 |
| Average slope (degrees) | U | 14.476 | 4.4367 | 228.5 | 15.48 | 0.000 |
|  | M | 11.6 | 12.113 | -11.7 | -0.37 | 0.711 |
| Average altitude (m) | U | 936.21 | 871.95 | 80.8 | 6.77 | 0.000 |
|  | M | 918.96 | 941.42 | -28.3 | -0.95 | 0.348 |
| Average distance to Moyobamba (km) | U | 8.2455 | 7.9234 | 7.7 | 0.58 | 0.565 |
|  | M | 8.356 | 9.2213 | -20.6 | -0.76 | 0.441 |
| Total forest cover 2010 (ha/hh) | U | 5.5891 | 2.6165 | 58.8 | 4.27 | 0.000 |
|  | M | 3.9059 | 2.8726 | 20.5 | 1.10 | 0.275 |
| Δ Total forest 2005-2010 (ha/hh) | U | .65257 | .1178 | 43.9 | 2.99 | 0.003 |
|  | M | .53508 | .20703 | 27.0 | 0.89 | 0.377 |
| Household’ members 2010 | U | 2.7381 | 2.9476 | -19.8 | -1.11 | 0.266 |
|  | M | 2.8 | 2.6572 | 13.5 | 0.52 | 0.607 |
| Assets index 2010 | U | 2.4048 | 3.5939 | -56.9 | -3.64 | 0.000 |
|  | M | 2.6 | 3.0234 | -20.2 | -0.70 | 0.487 |
| Income 2010 (PEN) | U | 9489.8 | 17664 | -55.5 | -2.83 | 0.005 |
|  | M | 9016.7 | 11376 | -16.0 | -0.95 | 0.348 |

**S2K Table. Covariate balance achieved to compare T1 (*command-and-control* measures) vs control households groups using the R-PS matching algorithm.**

| **Covariate** | **Unmatched (U)/ Matched (M)** | **Mean** | | **% St. Dif.** | **t-test** | |
| --- | --- | --- | --- | --- | --- | --- |
|  |  | **Treated** | **Control** |  | **t** | **p > \|t\|** |
| Total size of managed lands (ha) | U | 6.6865 | 4.1945 | 38.3 | 2.33 | 0.020 |
|  | M | 4.4535 | 4.9109 | -7.0 | -0.23 | 0.823 |
| Average slope (degrees) | U | 16.2 | 4.4367 | 238.8 | 17.00 | 0.000 |
|  | M | 12.063 | 12.023 | 0.8 | 0.02 | 0.981 |
| Average altitude (m) | U | 912.08 | 871.95 | 54.8 | 4.38 | 0.000 |
|  | M | 922.19 | 929.96 | -10.6 | -0.26 | 0.795 |
| Average distance to Moyobamba (km) | U | 9.738 | 7.9234 | 83.1 | 3.93 | 0.000 |
|  | M | 9.6906 | 10.014 | -14.8 | -0.67 | 0.509 |
| Total forest cover 2010 (ha/hh) | U | 5.8047 | 2.6165 | 63.3 | 4.51 | 0.000 |
|  | M | 3.5972 | 4.2383 | -12.7 | -0.39 | 0.701 |
| Δ Total forest 2005-2010 (ha/hh) | U | .48723 | .1178 | 29.4 | 2.00 | 0.046 |
|  | M | .18724 | .60582 | -33.4 | -1.17 | 0.253 |
| Household’ members 2010 | U | 3.075 | 2.9476 | 10.7 | 0.64 | 0.521 |
|  | M | 3.25 | 2.8229 | 36.0 | 0.97 | 0.340 |
| Assets index 2010 | U | 2.625 | 3.5939 | -52.4 | -3.03 | 0.003 |
|  | M | 2.875 | 3.0447 | -9.2 | -0.31 | 0.761 |
| Income 2010 (PEN) | U | 8154.3 | 17664 | -66.2 | -3.13 | 0.002 |
|  | M | 8588.3 | 8030 | 4.0 | 0.19 | 0.853 |

**S2L Table. Covariate balance achieved to compare T2 (*PES-ICDP* mix) vs control households groups using the R-PS matching algorithm.**

| **Covariate** | **Unmatched (U)/ Matched (M)** | **Mean** | | **% St. Dif.** | **t-test** | |
| --- | --- | --- | --- | --- | --- | --- |
|  |  | **Treated** | **Control** |  | **t** | **p > \|t\|** |
| Total size of managed lands (ha) | U | 6.2444 | 4.1945 | 32.5 | 1.98 | 0.049 |
|  | M | 4.4736 | 3.6145 | 13.6 | 0.76 | 0.451 |
| Average slope (degrees) | U | 14.476 | 4.4367 | 228.5 | 15.48 | 0.000 |
|  | M | 11.6 | 11.994 | -9.0 | -0.29 | 0.776 |
| Average altitude (m) | U | 936.21 | 871.95 | 80.8 | 6.77 | 0.000 |
|  | M | 918.96 | 940.91 | -27.6 | -0.92 | 0.360 |
| Average distance to Moyobamba (km) | U | 8.2455 | 7.9234 | 7.7 | 0.58 | 0.565 |
|  | M | 8.356 | 9.1343 | -18.6 | -0.68 | 0.500 |
| Total forest cover 2010 (ha/hh) | U | 5.5891 | 2.6165 | 58.8 | 4.27 | 0.000 |
|  | M | 3.9059 | 3.0535 | 16.9 | 0.88 | 0.384 |
| Δ Total forest 2005-2010 (ha/hh) | U | .65257 | .1178 | 43.9 | 2.99 | 0.003 |
|  | M | .53508 | .2236 | 25.6 | 0.84 | 0.405 |
| Household’ members 2010 | U | 2.7381 | 2.9476 | -19.8 | -1.11 | 0.266 |
|  | M | 2.8 | 2.6065 | 18.3 | 0.71 | 0.484 |
| Assets index 2010 | U | 2.4048 | 3.5939 | -56.9 | -3.64 | 0.000 |
|  | M | 2.6 | 3.0194 | -20.1 | -0.70 | 0.490 |
| Income 2010 (PEN) | U | 9489.8 | 17664 | -55.5 | -2.83 | 0.005 |
|  | M | 9016.7 | 11135 | -14.4 | -0.87 | 0.391 |
