# Supplementary material for: Adding rewards to regulation: The impacts of watershed conservation on land cover and household wellbeing in Moyobamba, Peru
Source: PLoS One. 2019 Nov 20;14(11):e0225367. doi: 10.1371/journal.pone.0225367 (PMC6867640; doi:10.1371/journal.pone.0225367)
Supplement: S3 Table — (DOCX) [file pone.0225367.s003.docx]

**S3 Table. Additional estimations.**

**S3A Table. Intra cluster correlations (ICC).** We checked for spillover effects as all ICC are lower than 0.1, that is to say that variance across sectors explain less than 10% of the total variation in all outcomes.

|  | **Outcomes** | | | | | |
| --- | --- | --- | --- | --- | --- | --- |
|  | **Plot dataset** | | **Household dataset** | | | |
|  | **Δ Prim. for. change** | **Δ Sec. for. change** | **Annual income change** | **Assets change** | **Improved life quality** | **Worsened life quality** |
| Intra cluster corr. (Rho) | 0.000 | 0.000 | 0.094 | 0.089 | 0.083 | 0.01 |
| SE | (0.000) | (0.008) | (0.049) | (0.054) | (0.054) | (0.023) |

**S3B Table. Association matrix among wellbeing outcomes.** Association between households’ incomes and assets were estimated using the Pearson’s pairwise correlation. To measure association between (semi) continuous variables and binary ones (the two perceived wellbeing variables) we ran probit regressions using each binary variable as only regressor, and marginal effects at means are reported. Finally, to measure the association between both binary variable we used the tetrachoric correlation coefficient [-1, 1]. We report statistical significance at 10%*, 5%** or 1%***.

|  | **Year HH. Income change** | **Assets** | **Improved life quality (0/1)** | **Worsened life quality (0/1)** |
| --- | --- | --- | --- | --- |
| **Year HH. income change** | 1.00 | 0.157* | -0.02 | 0.005 |
| **Assets** | - | 1.00 | 0.06 | -0.036 |
| **Improved life quality (0/1)** | - | - | 1.00 | -1.00*** |
| **Worsened life quality (0/1)** | - | - | - | 1.00 |

**S3C Table. Estimation of the inflation-adjusted updated costs of the initiative.** We share each project annual amounts in equal percentages according its corresponding lifespan (Fig 1), because we don’t access to information detailed timing of each disbursement. We use 4% as discount rate following public investment National regulations in force*, and an exchange rate of 3.3 PEN per US dollar.

| **Project name** | **0** | **1** | **2** | **3** | **4** | **5** | **6** | **7** | **8** | **9** | **10** | **11** | **12** |
| --- | --- | --- | --- | --- | --- | --- | --- | --- | --- | --- | --- | --- | --- |
|  | **2004** | **2005** | **2006** | **2007** | **2008** | **2009** | **2010** | **2011** | **2012** | **2013** | **2014** | **2015** | **2016** |
| Cuencas Andinas | 109,000 | 109,000 | 109,000 |  |  |  |  |  |  |  |  |  |  |
| Proyecto Especial Alto Mayo - PEAM |  |  |  |  |  | 386,223 | 386,223 | 386,223 | 386,223 |  |  |  |  |
| EPS Moyobamba |  |  |  |  |  |  |  | 188,000 | 188,000 | 188,000 |  |  |  |
| RARE-Pride Campaign |  |  |  |  |  |  |  |  | 98,000 | 98,000 |  |  |  |
| BID-MINAM |  |  |  |  |  |  |  |  |  |  | 132,110 |  |  |
| Profonanpe |  |  |  |  |  |  |  |  |  |  | 32,500 | 32,500 |  |
| **Annual cash flow** | **109,000** | **109,000** | **109,000** | **0** | **0** | **386,223** | **386,223** | **574,223** | **672,223** | **286,000** | **164,610** | **32,500** | **0** |
| Annual nationwide average inflation | 3.70% | 1.60% | 2.00% | 1.80% | 5.80% | 2.90% | 1.50% | 3.40% | 3.70% | 2.80% | 3.20% | 3.50% | 3.60% |
| **Adjunted annual cash flow** | **109,000.00** | **107,283.46** | **104,767.40** | **0.00** | **0.00** | **334,781.35** | **353,217.23** | **454,398.42** | **502,670.40** | **223,063.24** | **120,132.15** | **22,260.74** | **0.00** |
| **Updated costs of the initiative** | ***S/2,198,073.97*** | | **$666,083.02** | |  |  |  |  |  |  |  |  |  |
| **Inflation-adjusted updated costs of the initiative** | ***S/1,828,279.29*** | | **$554,024.03** | |  |  |  |  |  |  |  |  |  |

(*) <http://www.minam.gob.pe/oficina-general-de-planeamiento-y-presupuesto/wp-content/uploads/sites/139/2018/01/29.09.2017-Directiva-002-II-parte-MEF-1.pdf>.
